# Editorial: Current state of the art of human brain white matter: From structural and functional connectivity to neurosurgical applications

**DOI:** 10.3389/fneur.2022.1068212

**Published:** 2022-11-22

**Authors:** Emanuele La Corte, Edgar G. Ordóñez-Rubiano, Wellingson Silva Paiva, Jason Michael Johnson, Graziano Serrao

**Affiliations:** ^1^Department of Neurosurgery, Fondazione IRCCS Istituto Neurologico Carlo Besta, Milan, Italy; ^2^Department of Biomedical and Neuromotor Sciences (DIBINEM), Alma Mater Studiorum University of Bologna, Bologna, Italy; ^3^Department of Neurological Surgery, Fundación Universitaria de Ciencias de La Salud (FUCS), Hospital de San José, Bogotá, Colombia; ^4^Neurosurgery Division, Department of Neurology, Clinical Hospital, Faculdade de Medicina da Universidade de São Paulo (FMUSP), São Paulo, SP, Brazil; ^5^Department of Diagnostic Radiology-Neuro Imaging, The University of Texas M.D. Anderson Cancer Center, Houston, TX, United States; ^6^San Paolo Medical School, Department of Health Sciences, Università Degli Studi di Milano, Milan, Italy

**Keywords:** anatomy, central nervous system, connectome, functional mapping, hodology, human brain, meta-network, white matter tracts

Recent knowledge of brain white matter anatomy represents an important milestone in modern neuroscience and neurosurgery development (1, 2). The human brain anatomy study recently shifted from a grey-matter-centered approach to a more integrative study of functional white matter connections (3–5). Auburtin, Bouillaud, and Broca, as “*localizationists,”* based their landmarks physiological studies on specific cortical regions; whereas, Wernicke, Liepmann, and Dejerine, as “*connectionists,”* were interested in the analysis of the human brain connections and developed a network theory where multiple and simultaneous transmissions of information could represent the backbone of brain function (6, 7). During recent times, neurosurgery shifted from a traditional planning strategy focused on a purely lesional topography toward a meta-network perspective and evidence of newer network-based circuitopathies (8–10). This paradigmatic shift enhanced neurosurgery to perform extensive resections in supposedly “inoperable” regions and to develop tailored programs of neurological, cognitive, and behavioral rehabilitation, aiming for the preservation of patients' quality of life (8, 11). Complex brain functions are executed through the synchronization between different cortical epicenters (“*nodes”*), which are connected through bundles of white matter (“*edges”*), to ensure a dynamic interaction between parallel delocalized subnetworks (4). The chance to non-invasively examine the human brain connections and the integration of multiple imaging approaches will hopefully provide new metrics about the functional organization of the nervous system to be incorporated into neurosurgical applications (12, 13). These include advanced techniques of brain mapping such as direct cortical and subcortical stimulation and integrative neuropsychology to disclose brain networks in neurosurgical patients; advanced pre- and post-operative neuroimaging (such as DTI, DSI, fMRI, resting-state fMRI, MEG, TMS) and anatomical dissection techniques of human brain white matter (10, 14).

The present Research Topic aims to collect the current advancements in human brain white matter anatomy and function in perioperative stages to tailor cognitive rehabilitation to maximally preserve patient quality of life. Moreover, it will focus on the current state-of-art in brain white matter structural and functional connectivity applied to neurosurgery.

In his opinion paper “*Neural Connectivity: How to Reinforce the Bidirectional Synapse Between Basic Neuroscience and Routine Neurosurgical Practice?,”* Duffau stimulates the future neurosurgical community to augment the synergy between the clinical and basic sciences environments to provide a comprehensive translational view of brain architecture. The extensive application of electrostimulation mapping and accurate neuropsychological assessment in connectome-based surgery, together with a more sophisticated functional neuroimaging, revolutionized the concept of the brain organization from a static and localized system into a more dynamic one constituted by plastic neural pathways acting as a meta-network (15, 16). This translational approach during neurosurgical procedures could also guide the monitoring of high-order cognitive functions such as mentalization. Monticelli et al. provided a narrative review, “*Where We Mentalize: Main Cortical Areas Involved in Mentalization,”* elucidating the fundamental brain cortical areas and connections underpinning mentalization processes that could be assessed and preserved during neuro-oncological surgical procedures. The modern neuro-oncological surgical philosophy relies on preserving the functional and quality of life of patients as much as possible and a connectome-based approach may help to preserve, not only the classical motor and language functions, but also more complex neurocognitive ones. In the article “*Reducing the Cognitive Footprint of Brain Tumor Surgery,”* Dadario et al. provided a case-based review to better understand post-operative cognitive outcomes and to provide a guide on how to use connectomic information to reduce cognitive morbidity following brain surgery and optimize the post-operative neurorehabilitation. Motomura et al., “*Supratotal Resection of Gliomas with Awake Brain Mapping: Maximal Tumor Resection Preserving Motor, Language, and Neurocognitive Functions,”* presented a review on the supra-total resection of gliomas through the integration of intraoperative mapping in awake surgery. To augment the value of pre-operative neuroimaging studies, Tamura et al., “*Combining Pre-operative Diffusion Tensor Images and Intraoperative Magnetic Resonance Images in the Navigation Is Useful for Detecting White Matter Tracts During Glioma Surgery*,” proposed the combination of the pre-operative fractional anisotropy maps and intraoperative MR images into a neuronavigation system to aid in the localization of critical white matter pathways during glioma awake surgery. The continuous study of anatomy through *ex-vivo* dissection, as described by Joseph Klinger, represents a precious activity for the neurosurgeon to improve the knowledge of brain white matter tridimensional structure to provide less morbid surgery (17, 18). Dziedziec et al., “*Cortical and Subcortical Anatomy of the Parietal Lobe From the Neurosurgical Perspective,”* performed an anatomical study based on Klinger's technique on the parietal lobe and provided some neurosurgical perspectives on the different surgical trajectories to approach intra-axial lesion and their relationships to the arcuate and superior longitudinal fascicles. González-López et al., “*Cadaveric White Matter Dissection Study of the Telencephalic Flexure: Surgical Implications,”* performed an anatomical study and a comprehensive embryological review on the telencephalic flexure and provided some case examples of surgery within and around it. The accurate knowledge of subcortical connectivity improved the preservation of cognitive function of patients undergoing neuro-oncological and disconnective epilepsy surgeries. Janelle et al., “*Superior Longitudinal Fasciculus: A Review of the Anatomical Descriptions with Functional Correlates,”* performed a review summary on the different used eponyms of superior longitudinal fasciculus and highlighted the uncertainty, based on the actual technology, to intercept the different components of SLF and differentiate from the arcuate fasciculus. The advancement and the integration of different imaging techniques such as diffusion imaging techniques, and functional MRI in neuroscience applied to senescence and cleft palate surgery have been highlighted in two different studies (Derbie et al., “*Functional and Structural Architectures of Allocentric and Egocentric Spatial Coding in Aging: A Combined DTI and fMRI Study”*; Rao et al., “*Random Network and Non-rich-club Organization Tendency in Children With Non-syndromic Cleft Lip and Palate After Articulation Rehabilitation: A Diffusion Study”*). The cerebellum has been classically involved in movement coordination and its anatomical architecture is sometimes neglected. Recent evidence showed that the cerebellum is also involved in cognitive and behavioral functions. De Benedictis et al., **“***Networking of the Human Cerebellum: From Anatomo-Functional Development to Neurosurgical Implications,”* provided a comprehensive review of the current evidence on the anatomical and functional organization of the cerebellar connectome. The knowledge of the involved cerebellar networks in the neurocognitive process could improve the quality of approaches to reduce postoperative surgical comorbidity such as posterior fossa syndrome. The dynamic nature of reconstructing the neural brain networks resides in its ability to compensate the function in response to some stimuli (19). The presence of neuroplasticity in frontal glioma and after surgery violating the corpus callosum in colloid cyst removal is highlighted in two different studies (Ciavarro et al., “*Structural Brain Network Reorganization Following Anterior Callosotomy for Colloid Cysts: Connectometry and Graph Analysis Results”;* Mitolo et al., “*Neuroplasticity Mechanisms in Frontal Brain Gliomas: A Preliminary Study”*).

In conclusion, the present Research Topic includes comprehensive studies summarizing the current state of the art of cortical and subcortical human brain organization applied to neuroscience and neurosurgery. The papers depict the modern knowledge of neuroplasticity mechanisms, white matter anatomical pathways, and functional networks to be incorporated into routine neurosurgical practice. We hope for the future development of new artificial intelligence algorithms that could be able to reduce the subjectivity in the ROIs definition process. This would better qualitatively and quantitively discriminate the contribution of each different source or target structure in the intermingling connection tracts areas (i.e., dentate-rubro-thalamic tract) (20). We also hope this Research Topic will stimulate the neuroscientific and neurosurgical community into a deep and collaborative study of human brain white matter anatomy, to provide high-definition diagnostics and connectome-based neurosurgery oriented to the preservation of networks subserving high-order cognitive functions.

## Author contributions

EL, EO-R, WP, JJ, and GS: conceptualization, collection of the data, writing of the draft, and revision of the final manuscript. All authors are accountable for the content of the work.

## Conflict of interest

The authors declare that the research was conducted in the absence of any commercial or financial relationships that could be construed as a potential conflict of interest.

## Publisher's note

All claims expressed in this article are solely those of the authors and do not necessarily represent those of their affiliated organizations, or those of the publisher, the editors and the reviewers. Any product that may be evaluated in this article, or claim that may be made by its manufacturer, is not guaranteed or endorsed by the publisher.
